# Fatigue and Fracture Behavior of Composite Materials

**DOI:** 10.3390/ma16237292

**Published:** 2023-11-23

**Authors:** Davide Palumbo, Rosa De Finis

**Affiliations:** 1 Department of Mechanics, Mathematics and Management (DMMM), Politecnico di Bari, Via Orabona, 5, 70125 Bari, Italy; davide.palumbo@poliba.it; 2 Department of Engineering of Innovation, Università del Salento, Ecotekne Campus, Via per Monteroni, 73100 Lecce, Italy

## 1. Introduction

Presently, composites are one of the top-of-the-range materials used in different industrial sectors and represent the best alternative to metal alloys in those applications where higher mechanical properties and lower weights are required.

Composites are, by their nature, lightweight and resistant materials. The possibility of tailoring their properties in a synergistic fashion to meet a wide range of mechanical performances makes composites an interesting alternative for achieving consistent weight savings in primary structures. However, these positive aspects are paid by the drawbacks of multiple and complex damage mechanisms and an overall brittle behaviour making the study and prediction of fatigue and fracture mechanics behaviours challenging.

As the composites industry develops new materials and processes, a fundamental issue that needs to be addressed is how designers will adapt to the opportunities that composites provide, accounting for the specific damage mechanisms and response of the material.

In effect, certain guidelines are required for designers to understand damage mechanics, accumulation and mechanisms, and the complex failure behaviour of structures, with the aim of predicting the structural response, relying on structural health monitoring that supports a damage-tolerance approach.

In this way, the use of experimental techniques is fundamental to enhance the material response assessment under loading. Techniques like thermography or acoustic emissions or digital imaging correlation are useful to gain information about damage onset and localisation. Moreover, they provide different parameters to define damage models aimed at predicting the material residual life under specific loading conditions.

In this Special Issues, recent advances in the research on these topics are presented, particularly aiming to show:

New structural functions (e.g., composite patches for repairing cracked structures);

New methodologies for the processing of data acquired from different sensors with the aim of assessing parameters to study the damage behaviour of the material.

In dealing with these challenging aspects of composite material behaviour assessment, the goal of the present Special Issue is to improve the current knowledge on mechanical performance, damage processes, and design rules for proposing new testing and analysis paradigms.

## 2. Contributions

In the present Special Issue, the contributions can be divided into different topics: those aimed at modelling the composite behaviour [1,2], those aimed at evaluating the material response via classic [3,4] and innovative methodologies involving the use of experimental techniques [5,6,7], and one focused on presenting new functions for composite repair [8].

The role of the modelling of mechanical behaviour is crucial for defining those variables affecting the fatigue and fracture mechanics behaviour. In the work of Li et al. [1], accounting for the complexity of the moulding process of braided composites producing a variability of the internal geometry (yarn path with uncertainty), that in turn affects the fatigue-resistance properties, an uncertainty modelling method was proposed to investigate the influence of the randomness of the yarn path on mechanical properties. The statistical characteristics of the equivalent elastic and thermal parameters were predicted using a combined approach: software simulation and finite element analysis software. In this way, the authors implemented in a rapid way a meso-uncertainty model and studied the mechanical properties of composites in a more efficient way.

The presence of voids and pores affects the fatigue response of materials, and it is important to have a model that simulates the phenomenon as close as possible to the physics. In the work of Mu et al. [2], a seepage model based on smoothed particle hydrodynamics (SPH) was developed for the seepage simulation of pore water in porous rock mass media. The adopted theoretical methodology led to the development of an extended hydro-mechanical coupling model to simulate the crack propagation and coalescence process of rock samples with prefabricated flaws undergoing hydraulic and compressive loads. The model was validated using experimental data, and the results showed that with the increase in flaw water pressure, the crack initiation angle and stress of the wing crack decreased gradually.

Experimental characterisation is the common denominator between studying material response and validating theoretical and numerical fatigue models. In the work of Santos et al. [3], the behaviour of carbon laminates nano-reinforced with CNFs was explored under different testing conditions (bending tests and interlaminar shear tests in different environments such as hydrochloric acid, sodium hydroxide, water, and temperature). The obtained results demonstrate the relevant improvements in bending stress and bending stiffness due to the incorporation of CNFs. Referring to aggressive environments, the corrosive one significantly affects the ILSS response; however, in the presence of CNFs added to the resins, a decrease in ILSS was observed in comparison with the laminates produced with neat resin. In general, the authors found that the addition of CNFs was beneficial as it reduced the degradation of the laminates.

The work of Calabrese et al. [4] took into consideration a hybrid PBO-Glass FRCM composite composed of one layer of textile embedded between two layers of a cement-based matrix composite; in particular, the aim was to evaluate the effect of low- and high-cycle fatigue on tensile properties, namely the tensile strength, ultimate tensile strain, and secant modulus. This work provides important indications on the tensile behaviour of PBO FRCM composite coupons subjected to high- and low-cycle fatigue tests. The results of HCF showed that the axial strain and energy dissipated had a stable phase during the test and a rapid increase during the final unstable fatigue phase, while the axial stiffness decreased following an approximately linear trend during the entire fatigue test. The LCF results showed that the axial stiffness decreased during the first cycles and then tended to stabilise to a constant value; similarly, the energy dissipated decreased during the first cycles and generally approached an approximately constant value during the following cycles.

In the work of De Giorgi et al. [5], the fatigue tests on composites were assisted by an infrared detector. In this research, the temperature represented an index to evaluate the fatigue damage as a function of the number of cycles for unidirectional CFRP made by RTM technology. The authors found a direct correlation between the damage index corresponding to 90% of the fatigue life and the temperature variation in the most stressed area.

A different analysis of the thermal signal, in the frequency domain, was presented by Shiozawa [6] and De Finis [7].

These two works aimed to study the temperature harmonic components related to thermoelastic temperature variations (reversible temperature changes) and dissipative temperature variations (from irreversible heat sources). In particular, the work of Shiozawa et al. [6], on short fibre-reinforced plastics (SFRPs), aimed to detect delamination defects. In their work, Shiozawa analysed the thermoelastic-related and dissipative-related amplitude harmonics components of thermal signal, and the author found a distortion of the second harmonic component possibly caused by a change in the stress-sharing condition between the fibre and resin due to delamination damage. Following this approach, the second harmonic of the temperature component can be understood as a useful parameter for the detection of delamination defects and the evaluation of damage propagation. On this latter topic, De Finis et al. [7] presented a correlation between the second harmonic component of the thermal signal and the area of the hysteresis loop during constant amplitude fatigue tests on CFRP. The area of hysteresis loop is a well-established parameter to estimate the damage in a material as it represents the energy involved in irreversible fatigue processes. The second harmonic temperature component is related to the twice increase (in one loading cycle) in the deformation; hence, it is related to dissipative phenomena. The potential of the approach is to use second harmonic temperature maps to estimate the energy dissipations, then if stress is known, it would be possible to reconstruct the S/N curve. Under a qualitative point of view, the use of thermography [5,6,7] allows for the damage to be localised and possibly the generating mechanism to be identified.

In this scientific field in recent years, a change has been observed in conceiving composite materials not only for bulk structural purposes but also for repairing functions, due to the development of more flexible and effective manufacturing processes. In this step forward, Yousefi et al. [8] treated the composite patches exhibiting unique properties such as lightness, ease of application, high flexibility, and high stiffness that are gaining increasing interest in industrial applications, such as aerospace structures and automobile industries. One of the most important features of composite patches is that, unlike traditional methods, they do not cause any damage to the parent plate and can be replaced several times in a more sustainable fashion. The results presented in [8] show the positive effects on the fatigue life of a cracked aluminium component repaired with a composite patch.

## 3. Conclusions and Outlook

The present Special Issue focused on collecting new researchers showing the recent steps taken forward on new advanced methods or techniques for the testing, analysing, and monitoring of composite behaviour during fatigue loadings and fracture mechanics and to shed light on the structural response of newly developed composites or new more functional composite applications.

The presented researchers demonstrate on the one hand the great capability of composites to respond to structural needs, especially in fatigue applications; on the other hand, these findings represent an important tool for conveying new research toward more functional (from a structural point of view) and more sustainable composites.

Furthermore, the use of experimental techniques demonstrates that structural monitoring with a proper experimental technique (like thermography) allows for the problems related to damage and the sudden failure of these materials due to the possibility of an early damage assessment to be overcome.

Future development will be in the direction of studying new damage parameters for structural health monitoring and finding new strategies to fully exploit the structural potential of these materials to prevent or contain fatigue and fracture processes.

Finally, the editors would like to thank all the reviewers for their time spent reviewing these manuscripts and providing valuable opinions and suggestions. The editors also wish to thank Ms. Hellen Hu and the editorial staff for their support in the following-up, peer review, and publication process.
